# Method of Depriving Quinine of Its Bitterness

**Published:** 1850-08

**Authors:** 


### MATERIA MEDICA AND THERAPEUTICS.

*Method of depriving Quinine of its Bitterness.*—Believing that I have discovered a method by which quinine may be quite deprived of of its great bitterness, without injuring its virtues in the least, I wish to make it known to the profession. Perhaps I have been anticipated, but if it be so I am not aware of it.

In August last, having occasion to prescribe for a little patient, who was affected both with diarrhœa and intermittent fever, I ordered a combination of quinine and tannic acid. The child took it so readily that I tasted it, and was surprised to discover no taste of quinine, which I at once attributed to the combination.

I have since prescribed it in a number of instances, and found that whilst it was equally effectual, it was far more palatable than any other combination of quinine I was acquainted with. On referring to the American Journal of Medical Sciences, Vol. xix., page 219, (1836,) it will be found that Dr. Ronander, Secretary of the Swedish Medical Association, recommended the tannate of quinine and cinchonin as the most active ingredients of the Peruvian bark. He asserts that he has cured, by their means, several cases of obstinate ague, which had resisted the use of sulphate of quinine, and other powerful remedies. Nothing is said in the extract from the original paper in Hecker's Annals, December, 1834, of the taste of the tannate of quinine. Compared with the sulphate, it is almost tasteless.

The following is the extemporaneous prescription I am in the habit of ordering for a child two years old —

R. Quiniæ sulph. gr. x.;

Acid. tannici gr. ij.;

Aquæ, ℥vj.;

Syrup aurant. ℥ij. M.

A teaspoonful every hour or two.

I enclose a note on the subject from one of our most intelligent and careful apothecaries:—

*Dear Sir,*—I find, after trying a number of times, combinations of quiniæ sulphas and acidi tannici, in different proportions, that ten grains may be deprived of its bitterness in a great degree by the addition of one grain and a half of tannic acid. I think this is a proper proportion.

J. V. D. Stewart.

*Dr. Thomas of Baltimore in American Journal of Medical Science.*

